# Choosing a Journal for Submission: Don’t Fall Prey

**DOI:** 10.14797/mdcvj.650

**Published:** 2021-09-24

**Authors:** Barbara Gastel

**Affiliations:** 1 Texas A&M University, College Station, Texas, US

**Keywords:** predatory journals, fraudulent journals, scholarly communication

## Abstract

The column in this issue is supplied by Barbara Gastel, MD, MPH, who is professor of veterinary integrative biosciences and of humanities in medicine at Texas A&M University, where she coordinates the graduate program in communicating science. Dr. Gastel obtained her medical and public health degrees from Johns Hopkins University. She is first author of the current edition of How to *Write and Publish a Scientific Paper* (ABC-CLIO, 2016).

If it seems too good to be true, it probably is.

The situation is all too common. An early-career physician or scientist wishes to publish some recently completed research. But where to publish it? Someplace like the *New England Journal, JAMA*, or *Annals?* Or perhaps a more realistic target, such as a subspecialty journal?

As the prospective author ponders, an email message serendipitously arrives. It compliments the recipient on being an eminent researcher and invites them to submit an article for publication. According to the message, this journal publishes papers in many fields of science, medicine, and even humanities. It says submissions are published in less than a week. Its editorial board lists many prominent researchers—including some the prospective author thought had long ago retired. And the publication fee is less than $100, unlike the hundreds or thousands of dollars charged by many open access journals.

The author submits the paper to the journal, along with the fee. But the paper is just posted without peer review. Or maybe it never appears at all. What had seemed to be a journal turned out to be a fraud.

In recent years, a multitude of fraudulent journals—commonly called *predatory journals*—have arisen. These “journals” take authors’ money without providing the services of a valid journal, such as peer review and editing. They arose simultaneously with legitimate open-access journals, which, rather than charging for subscriptions, defray publication costs by charging authors fees. Some say so-called predatory journals are not truly predatory, as they capitalize on a market for those wanting to list publications among their credentials.^1^ But papers submitted to fraudulent entities are unlikely to reach their intended readers. They add no luster to a curriculum vitae and indeed may tarnish it.

How can one avoid falling prey to the lure of fraudulent journals? Some suggestions:

- Seek evidence that the journal is solid: Have you read papers from it? Do libraries carry it? Is it listed in *PubMed?* Not all good journals meet such criteria, but if the answer to such questions is “no,” one should investigate further.
- Consult those with expertise in such matters: Ask a mentor, or check with a medical librarian; the library community has taken a lead in identifying fraudulent journals.
- Look for clues that the journal may be less than a quality endeavor: Such clues can include grammatical errors in the invitation letter and on the journal website, lack of contact information on the website, and lack of papers posted.

Sources of further advice include a recent journal article presenting tips and resources for avoiding predatory journals,^2^ a webpage from The Ottawa Hospital Research Institute,^3^ and the website “Think.Check.Submit.,” which has a checklist for assessing whether a journal seems valid.^4^ This site also contains guidance on determining whether a book publisher is legitimate.

Indeed, those enticing emails can arrive not just from fraudulent journals but also from analogous shams regarding books and conferences. So, investigate critically. Don’t let “seems too good to be true” become “seems too bad to imagine.”
